# Optical Patterning in Photoresponsive Azobenzene-Based Waterborne Coatings

**DOI:** 10.1021/acsaom.2c00083

**Published:** 2022-11-17

**Authors:** Sterre Bakker, Esmee de Korver, Michel Fransen, Esra Kamer, Gerald A. Metselaar, A. Catarina C. Esteves, Albert P. H. J. Schenning

**Affiliations:** † Laboratory of Stimuli-responsive Functional Materials and Devices, Department of Chemical Engineering and Chemistry, Eindhoven University of Technology, P.O. Box 513, 5600 MB Eindhoven, The Netherlands; ‡ SyMO-Chem B.V., P.O. Box 513, 5600 MB Eindhoven, The Netherlands; § BASF Nederland B.V., Innovatielaan 1, 8447 SN Heerenveen, The Netherlands; ∥ Laboratory of Physical Chemistry, Department of Chemical Engineering and Chemistry, Eindhoven University of Technology, P.O. Box 513, 5600 MB Eindhoven, The Netherlands

**Keywords:** waterborne coating, azobenzene, photoresponsive, alkali-soluble resin, optical patterning

## Abstract

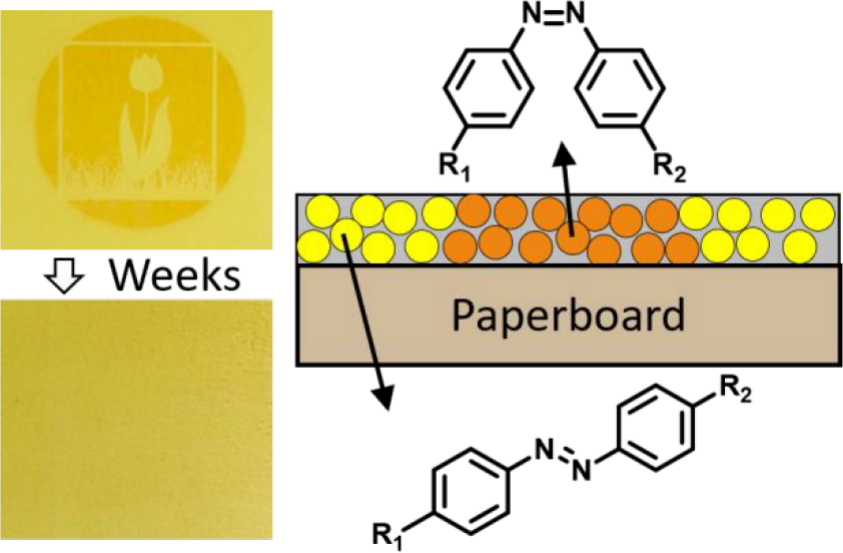

Reversible photoresponsive waterborne dispersions can be directly applied in the paint and printing industry and have several potential applications such as in rewritable optical patterns, security labeling, and sensing. However, the synthesis of such dispersions and application as waterborne coatings has rarely been reported. In this work, photoresponsive polymer dispersions containing light-responsive azobenzene were prepared by resin-stabilized emulsion polymerization. The aqueous dispersions consist of core/shell type polymer particles, where the resin forms the shell around the polymer core. The photoresponsive azobenzene was incorporated into the resin shell (azo-in-shell) or polymer core (azo-in-core). Waterborne coatings were easily prepared by applying the dispersions on glass or paperboard. The photoresponsive behavior of the azobenzene in the dispersions and coatings shows fast and reversible isomerization and a similar half-life of the *cis*-azobenzene regardless of its surrounding matrix in the coating, dispersion, or solution. Optical patterns were reversibly imprinted in the coatings on paperboard, which remain visible for several hours at room temperature and several weeks when stored in the freezer. The water barrier properties of the azobenzene-containing coatings on paperboard were unaffected by the addition of the azobenzene moieties and isomerization state. Our results reveal a class of photoresponsive azobenzene waterborne coatings that can be easily applied on different substrates.

## Introduction

A waterborne polymer dispersion or latex is a stable heterogeneous dispersion of polymer particles in water. It is a more environmentally friendly alternative to solvent-based systems as it allows the emissions of volatile organic compounds (VOCs) to be greatly reduced using waterborne dispersions. Usually, latexes are synthesized using emulsion polymerization of water-insoluble monomers, such as styrene and butyl acrylate, in the presence of surfactants or alkali-soluble resins (ASR). An ASR is an acid-rich resin that is water-soluble under alkaline conditions, and because it acts as the stabilizer during emulsion polymerization, it remains the shell around the polymer core.^1^ The resin-stabilized dispersion forms a coating when applied on a substrate, and during drying, both the water and the base evaporate.^2−5^ Due to the large difference in the glass transition temperature of the resin shell and polymer core, particle coalescence is (partially) hindered by the hard shell; as a result, the final coating consists of randomly distributed isolated polymer particles entrapped in the continuous matrix of the resin.^6−13^

Dynamic functionalities can be added to resin-stabilized waterborne dispersions by incorporating responsive molecules into the polymers. Such responsive waterborne dispersions change their properties upon exposure to a certain stimulus, such as light, pH, or temperature.^14−16^ The dispersion can, e.g., change color,^14−16^ or the polymer particles in the dispersion may aggregate and/or disaggregate.^17,18^ Potential applications for such photoresponsive polymer dispersions would be in making polymer films and/or coatings for rewriteable optical patterns, security labeling,^19^ or sensing.^15^ Light as a stimulus has a particular advantage in that it can be applied in a localized and untethered way. In photoresponsive polymer dispersions, the photochromic molecules undergo light-induced reversible changes in physical and/or chemical properties when illuminated with a specific wavelength.^20^ Two well-known and widely researched photochromic molecules are spiropyran^21^ and azobenzene^22^ derivatives, which exhibit different isomerization mechanisms, i.e., ring opening/closing and *E*/*Z* isomerization, respectively.

Several research groups reported waterborne photoresponsive polyurethane dispersions, which were prepared by adding azobenzene diols during the step-growth addition polymerization typically performed in an organic solvent, like acetone or tetrahydrofuran (THF). The photoresponsive polyurethane dispersion in water was then obtained upon blending with water and evaporation of the organic solvent. A color change was observed upon isomerization of the azobenzene upon illumination with ultraviolet (UV) light or in different acidic environments.^15,16,23,24^ Alternatively, photoresponsive dispersions have been synthesized by adding a reactive photoresponsive molecule during emulsion polymerization.^14,25^ For example, an acrylate-functionalized spiropyran was used during the emulsion polymerization of acrylate monomers.^14,26,27^ The ring-opening isomerization of the spiropyran in the dispersion resulted in a change in color from colorless to purple.^14,27^ Nonwaterborne azobenzene-functionalized materials have been reported for multiple applications.^28−35^ However, photoresponsive waterborne polymer dispersions are preferably prepared by emulsion polymerization as it is an efficient straightforward process that is easy to scale up, and the resulting dispersion can be directly used in the paint and printing industry. Furthermore, they have several potential applications such as in rewriteable optical patterns, security labeling, and sensing. Despite these advantages, such photoresponsive aqueous polymer dispersions have been sparsely reported or applied as waterborne coatings so far.

In our work, two approaches for preparing photoresponsive resin-stabilized polymer dispersions were investigated using azobenzene as the photochromic molecule. Because the polymer particles consist of two phases, azobenzene was incorporated into the poly(styrene/butyl acrylate) core (azo-in-core) or the resin shell (azo-in-shell). The isomerization behavior and kinetics of the azobenzene were investigated for both the dispersions and the coatings on glass. Optical patterns were imprinted in the coatings applied on paperboard by mask illumination of UV light and could also be reversibly erased by blue light exposure.

## Results and Discussion

**Figure 1:**
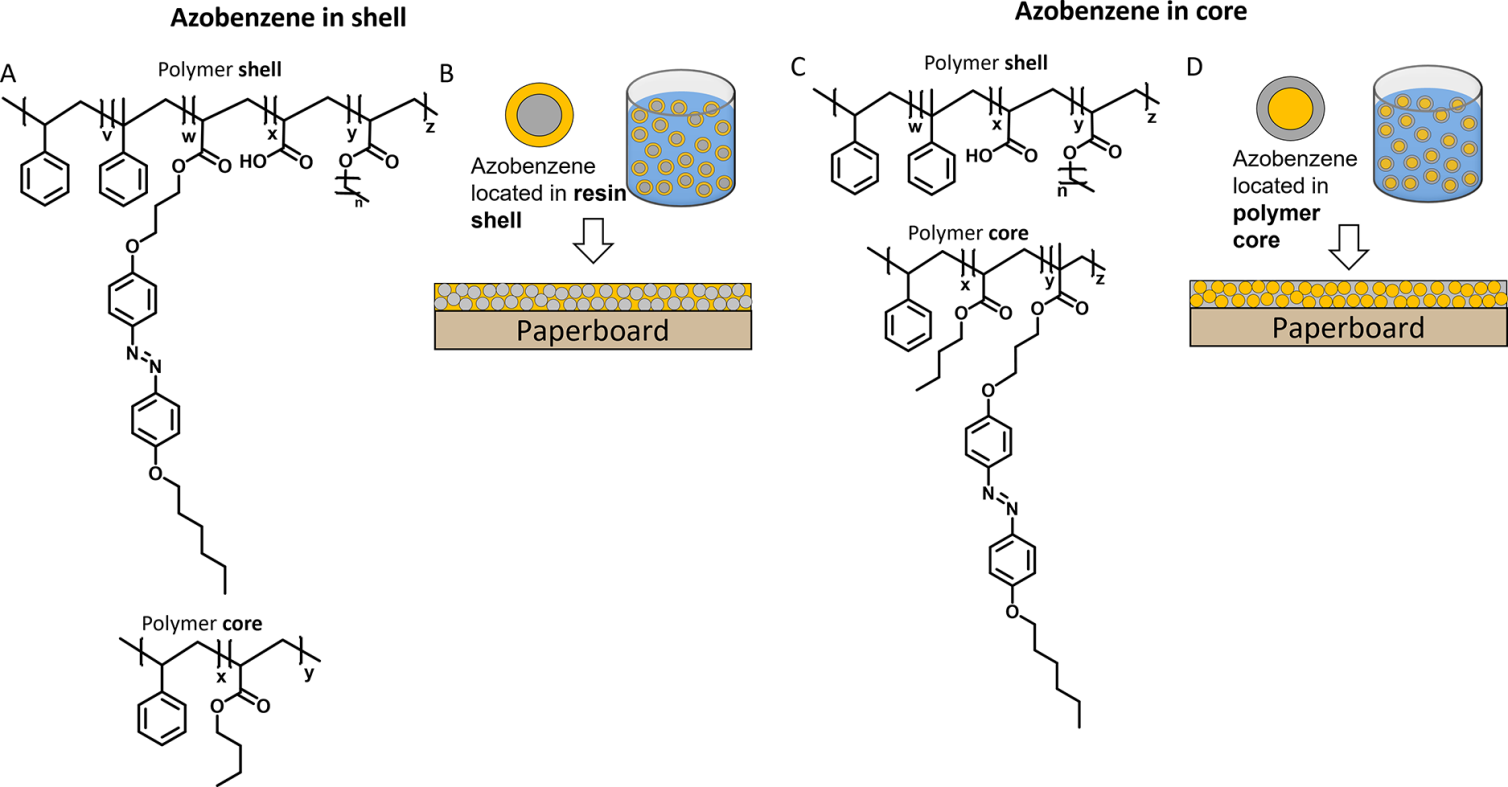
Chemical composition of the waterborne coatings where azobenzene was located (A) in the polymer shell or (C) in the polymer core. (B and D) Formation of the coating via application of the dispersion on paperboard.

**Figure 2:**
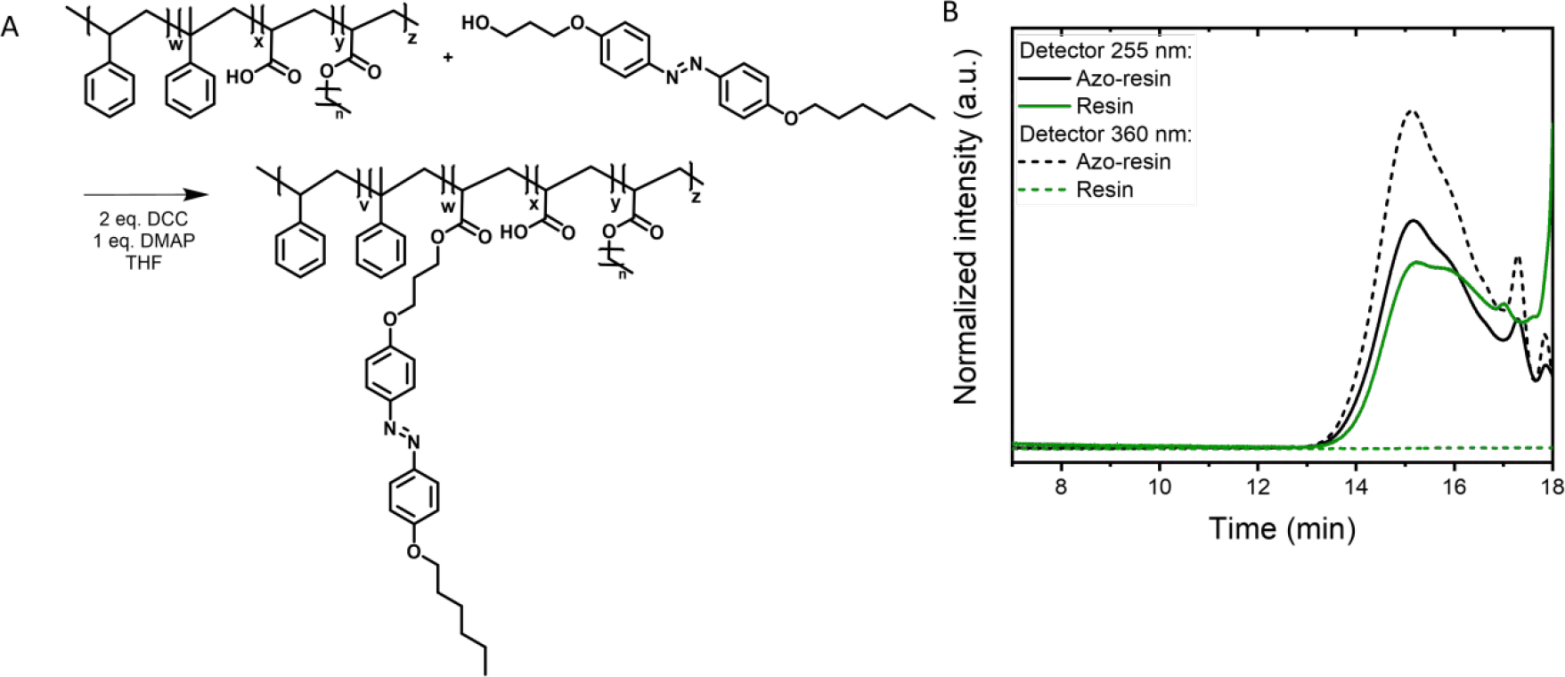
(A) Esterification of resin and alcohol-functionalized azobenzene under Steglich conditions. (B) GPC data of the azobenzene-functionalized resin and the initial resin as a reference. The detector was set to wavelengths (λ) 255 and 360 nm. The GPC profile was cut off at 18 min.

Two types of photoresponsive dispersions were prepared, namely, where the azobenzene was located in the resin shell [azo-in-shell (Figure 1A,B)] or in the polymer core [azo-in-core (Figure 1C,D)]. To incorporate the azobenzene into the resin shell (azo-in-shell), the resin needs to be functionalized with the photoresponsive azobenzene (Figure 2A). Therefore, 5% of the carboxylic acids in the resin were esterified with an alcohol-functionalized azobenzene derivative. The remaining carboxylic acid groups in the resin are used to ensure aqueous alkali solubility of the azobenzene-functionalized resin. The esterification was performed following a Steglich reaction.^36^ After purification, an orange glassy azobenzene-functionalized resin was obtained in a yield of 75%. The functionalized resin was characterized using gel permeation chromatography (GPC). The detector was set to wavelengths (λ) of 255 nm, where the aromatic units absorb, and 360 nm, where only the azobenzene derivative absorbs. Figure 2B shows the successful functionalization of the resin with the azobenzene as the spectra of both detectors overlap (solid and dashed black lines) and had a *M*_n_ of 4000 g/mol. The initial resin was measured as a reference and showed absorption only when the detector was set to 255 nm (Figure 2B, green lines). The two smaller peaks at higher retention times (∼17 min) originated from the azobenzene that reacted with smaller oligomers present in the initial resin. The degree of esterification was determined by using ^1^H nuclear magnetic resonance (NMR) with an internal standard, trimethylbenzene tricarboxylate, and UV–visible (UV–vis) analysis (see the Supporting Information). It was estimated that on average, the azobenzene-functionalized resin bears one azobenzene moiety per resin chain.

The azo-in-shell dispersion was prepared by semibatch emulsion polymerization of butyl acrylate and styrene, using the azobenzene-functionalized resin (*vide supra*) as a stabilizer. The dissolved resin and thermal initiator, ammonium persulfate (APS), were loaded into the reactor and heated to 84 °C, followed by slow addition of the monomer mixture to maintain starved feed conditions. The monomer conversion was low, which might be related to a small pH variation that can have an influence on the inhibition period of the polymerization reaction.^37^ Therefore, an additional redox initiator (70% aqueous *tert*-butyl hydroperoxide and sodium erythorbate) was also added to ensure polymerization. After polymerization, the reaction mixture was cooled and filtered. To prepare the azo-in-core dispersion, methacrylate-functionalized azobenzene (2 wt % of total solids) was mixed with the other monomers, styrene and butyl acrylate. The emulsion polymerization was performed following the same procedure. However, here only the APS thermal initiator was needed to achieve high monomer conversion. The obtained dispersions were characterized by dynamic light scattering (DLS), and the dry solid content was determined by gravimetry. The azo-in-core dispersion had a particle diameter of 115 nm, and the azo-in-shell dispersion had slightly larger particles (125 nm). The dry solid content was determined to be 43% for the azo-in-core and 37% for the azo-in-shell dispersions. The slightly lower dry solid content for the azo-in-shell dispersion can be explained by the addition of more water due to the addition of the second initiator. These properties are in agreement with reported characteristics of similar resin-stabilized dispersions prepared under comparable conditions.^38−41^

**Figure 3:**
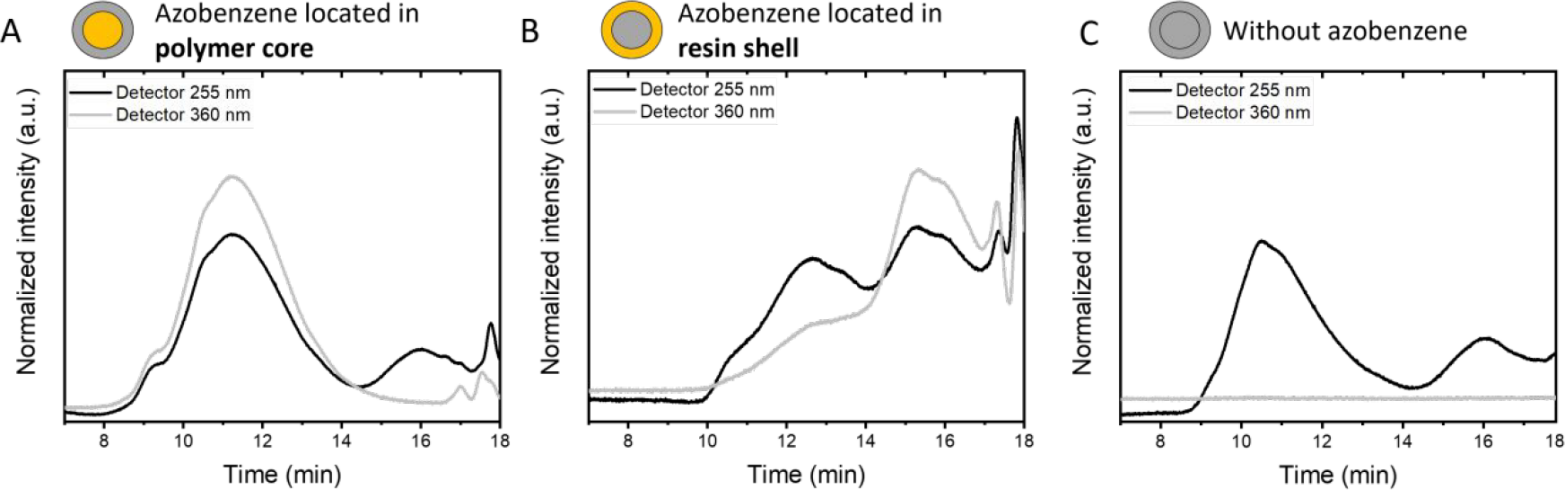
(A) GPC data of the azo-in-core film showing that the azobenzene is in the polymer core. (B) GPC data of the azo-in-shell film show that the azobenzene is mostly in the resin shell. (C) GPC data of the reference polymer film without azobenzene show two polymer fractions corresponding to the polymer core and resin shell. The two narrow small peaks at higher retention times represent the oligomers of the resin. The detector was set to wavelengths (λ) of 255 and 360 nm. The GPC profile was cut off at 18 min.

Both polymer dispersions were further characterized using GPC, for which the dried dispersion (film) was dissolved in THF as a solvent (Figure 3). For all dispersions, azo-in-core, azo-in-shell, and a reference without azo, two polymer fractions were observed at retention times of 11 and 16 min originating from the poly(styrene/butyl acrylate) core and the resin shell, respectively (Figure 3C). Figure 3A confirms that the azobenzene is indeed incorporated into the polymer core for the azo-in-core dispersion because the absorbance at 360 nm shows a clear fraction that matches with the retention time of the poly(styrene/butyl acrylate) core. For the azo-in-shell dispersion, Figure 3B shows that most of the azobenzene moieties are located in the resin shell; however, a small signal has also been observed at lower retention times. This indicates that approximately 20% of the azobenzene resin is incorporated into the polymer core. For both routes of the synthesis of the dispersions, a small portion of the resin appears to be grafted to the polymer core by chain transfer to resin or via reaction with residual terminal C=C bonds present in the resin.^42^ Because this grafted product will most probably end up buried in the polymer core fraction, this effect can be detected for only the azo-in-shell dispersion.

**Figure 4:**
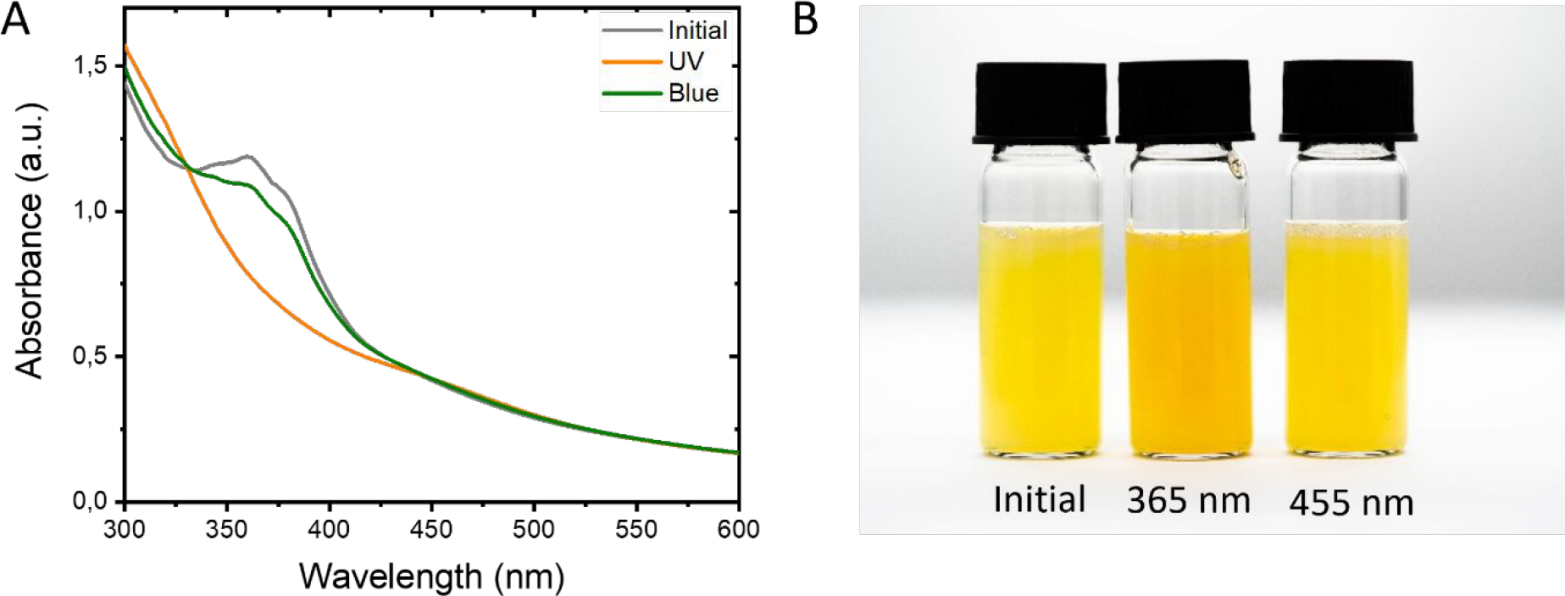
(A) UV–vis spectra show the light-induced isomerization of the diluted azo-in-core dispersion upon illumination with UV light (λ = 365 nm) and blue light (λ = 455 nm). (B) Photograph of the initial azo-in-core dispersion and after illumination with UV light (λ = 365 nm) followed by blue light (λ = 455 nm).

The photoresponsiveness of the azo-in-core dispersion was investigated by UV–vis spectroscopy as shown in Figure 4A. Before illumination, the UV–vis spectrum showed maximum absorbance at λ = 362 nm (gray line) characteristic of *trans*-azobenzene moieties. Scattering is observed for the shorter wavelengths, which is caused by the polymer particles in the dispersion. Illumination of the dispersion with UV light (λ = 365 nm) for 1 min results in *trans* to *cis* isomerization, which one can see by the disappearance of the absorbance at λ = 362 nm (orange line). This isomerization resulted in a change in color from yellow to orange as shown in Figure 4B. Subsequently, illumination with blue light (λ = 455 nm) triggered the back-isomerization from *cis* to *trans* and resulted in the restoration of the *trans*-azobenzene absorbance at λ = 362 nm (green line). However, the maximum absorbance was lower than the initial absorbance as *trans*-azobenzene also absorbs blue light (*vide infra*). The absorbance spectrum of the azo-in-shell dispersion was not included due to the large scattering. Also, the color was reversibly changed to yellow in time. The isomerization state of the azobenzene did not influence the particle diameter as determined by DLS (Figure S3).

**Figure 5:**
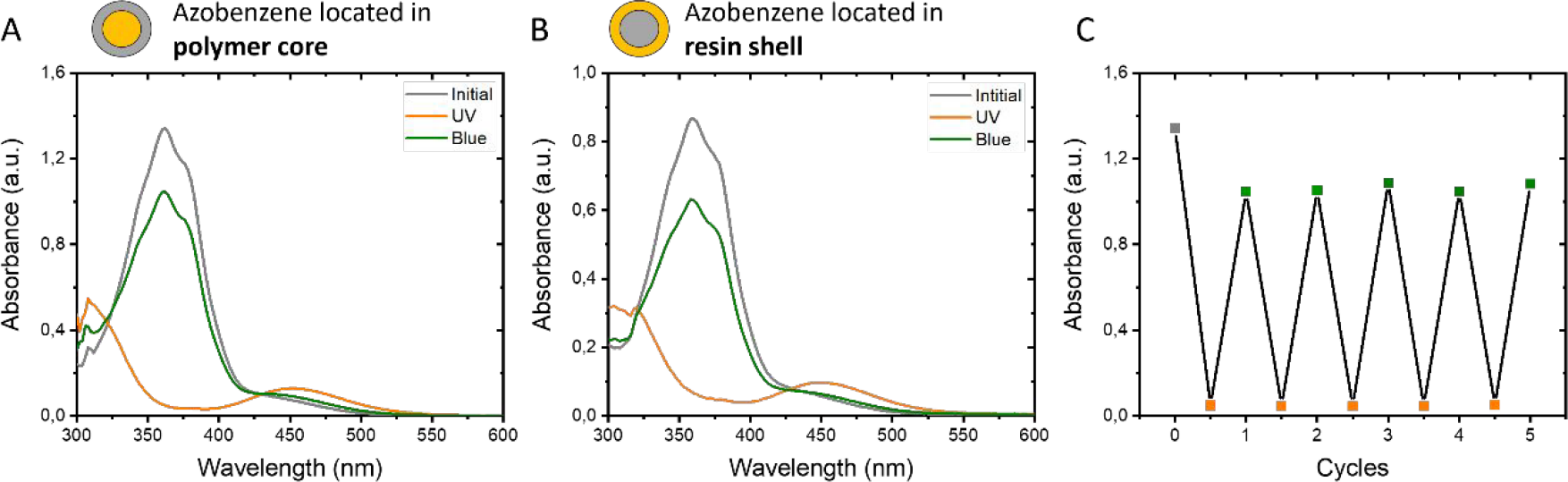
UV–vis spectra show the light-induced isomerization of the waterborne azobenzene-based coatings where azobenzene is incorporated into the (A) polymer core or (B) resin shell. The coatings were applied on glass. (C) The reversibility of the isomerization is shown by five cycles of illumination of the azo-in-core coating with UV (λ = 365 nm) and blue light (λ = 455 nm).

To study the isomerization of the azo-in-core and azo-in-shell coatings, the as-prepared dispersions were applied on glass using a gap applicator and dried on a hot plate at 60 °C for 1 h. Glass was used as it is a transparent and flat substrate. The width of the gap was chosen so that the maximum absorbance of the azobenzene in the coating is ∼1. Both coatings were transparent and did not show any scattering indicating that the azobenzene was well-distributed over the coating. Both azo-in-core and azo-in-shell initial coatings appeared yellow as their maximum absorbance is at λ = 362 nm (gray line, Figure 5A,B), which is at the same position as their respective diluted dispersions and the azobenzene starting compound dissolved in ethanol (Figure 4B and Figure S4A). This indicates that the formation of the film and the location of the azobenzene moieties in the dispersion (e.g., polymer core or resin shell) do not affect the absorbance of the azobenzene and that in both cases no aggregates are formed.

After illumination with UV light (λ = 365 nm) for 1 min, the *trans* configuration of the azobenzene isomerizes to the *cis* configuration, which has a maximum UV absorbance at λ = 452 nm (orange line). The isomerization resulted in a change in color of the coating from yellow to orange. A low UV light intensity was used to slow the *trans* to *cis* isomerization as one can see in Figure S6. Subsequent illumination of the coatings with blue light (λ = 455 nm) switched the azobenzene moieties back to their *trans* conformation, as evidenced by the absorbance at λ = 362 nm. This switch was accompanied by an observable change in color from orange to yellow. The small difference in absorbance at λ = 362 nm between the initial coating and the coating after illumination cycles can be attributed to the overlap between the absorbance of the *cis* isomer and that of the *trans* isomer at λ = 455 nm (*vide supra*). The *trans* isomer displays a very strong absorbance at λ = 362 nm. When illuminated with UV light, most azobenzene moieties are isomerized to the *cis* isomer, resulting in the disappearance of the absorbance at λ = 362 nm and a broad absorbance peak at λ = 452 nm. Under blue light illumination, the *cis* isomer is probed, resulting in *cis* to *trans* isomerization. Because a small absorption peak of the *trans* isomer is also present at that wavelength, however, the *trans* isomer is also excited, leading to incomplete recovery of the initial state. The isomerization of the azobenzene in the coating showed good reversibility for at least five illumination cycles as shown in Figure 5C.

The isomerization can be quantified with the half-life of azobenzene. The half-life is here defined as the time needed for the *cis*-azobenzene to isomerize to its *trans* isomer in which the maximum absorbance of the *trans* peak is half of the value of the initial *trans* peak before UV illumination. The half-life defines the stability of the *cis* isomer because the *trans* isomer is more thermodynamically stable and will eventually be formed without any external stimulus. The *cis* to *trans* isomerization can be described by a single-exponential decay shown in eq 1, where *N*(*t*) is the absorbance (unitless) at time *t* (hours), at a specific wavelength, *N*_0_ is the initial absorbance (unitless), and *t*_1/2_ (hours) is the half-life of the absorbance.
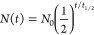
1

**Figure 6:**
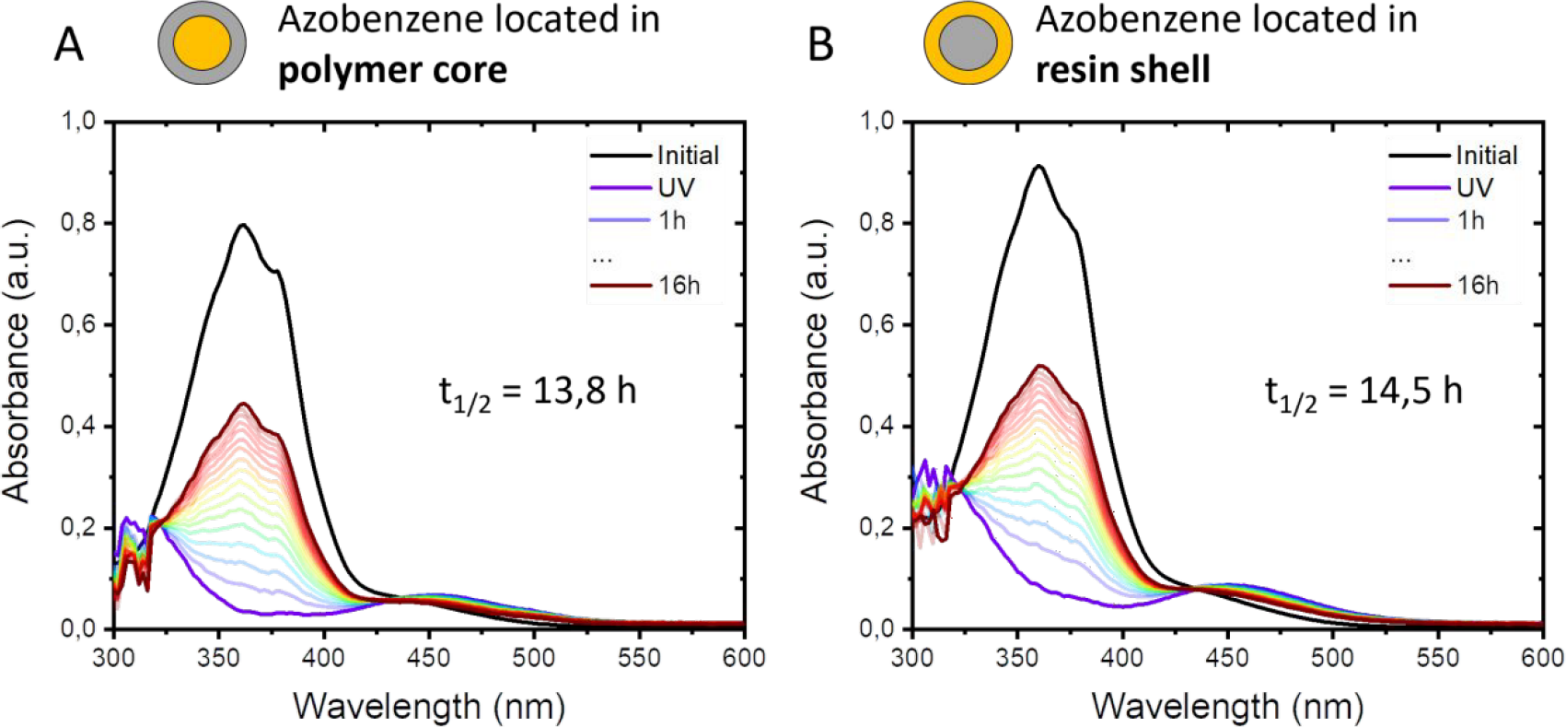
Half-life of the *cis*-azobenzene in the (A) azo-in-core and (B) azo-in-shell coatings applied on glass measured overnight at 25 °C.

The half-life of the *cis*-azobenzene incorporated into the polymer core was determined to be 13.8 h (Figure 6A). The half-life was slightly increased to 14.5 h when the azobenzene was incorporated into the resin shell (Figure 6B). The fits are shown in Figure S5. The glass transition temperature (*T*_g_) of the polymer core (24 °C) is much lower than that of the resin shell (90 °C) as determined in our previous work,^12^ which seems to have an only limited effect on the half-life of the azobenzene in the coatings. For comparison, the half-life of the azobenzene dissolved in ethanol was 12.8 h (Figure S4B), indicating that the surrounding polymer matrix plays a minor role in the isomerization kinetics.

To test the versatility of the photoresponsive dispersions, coatings were also prepared on white paperboard. Paperboard is a biobased material often used in food packaging. The as-prepared dispersions were applied on paperboard by bar-coating and dried at 60 °C for 1 h. The coatings on paperboard were characterized by atomic force microscopy (AFM) and attenuated total reflection Fourier transform infrared (ATR-FTIR) spectroscopy. The topography of the coatings showed randomly packed oval particles (Figure S7A). The spherical polymer particles appeared as an oval due to an artifact during the measurement. This topography originates from the hard shell/soft core polymer particles, in which the hard ASR shell prevents particle coalescence as explained in our previous work.^12^ The ATR-FTIR measurements show identical spectra for both coatings applied on paperboard (Figure S7B).

**Figure 7:**
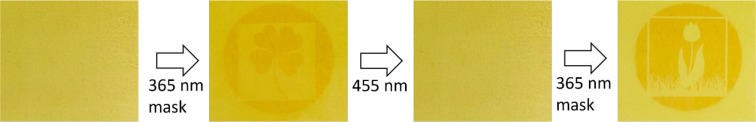
Photographs of the initial double azo-in-shell coating on paperboard, after mask illumination with UV light (365 nm) and blue light (455 nm), and after a second mask illumination with UV light (365 nm).

**Figure 8:**
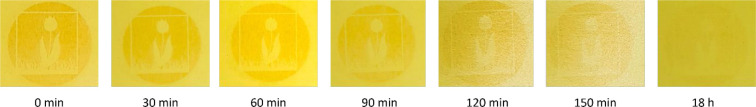
Photographs of the double azo-in-shell coating on paperboard after mask illumination with UV light (365 nm) show the pattern fading over time upon storage at room temperature.

**Figure 9:**
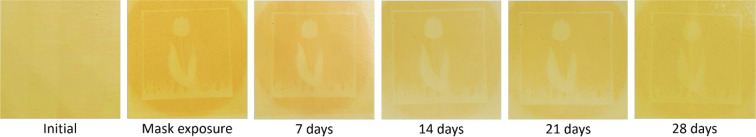
Photographs of the double azo-in-shell coating on paperboard after mask illumination with UV light (365 nm) show the pattern fading over time upon storage in a freezer.

To investigate the possible applications of the waterborne coatings, optical patterns were created. The optical patterns were imprinted by using mask illumination for double-coated paperboard as shown in Figure 7. The yellow coatings appeared to be scattering due to the application on a paperboard substrate. A second layer was applied to have a stronger initial yellow color as the concentration of azobenzene moieties in the coatings is low. The area exposed to UV light changed from yellow to orange due to *trans* to *cis* isomerization. The created pattern (clover) was subsequently erased by illuminating the entire coating with blue light, resulting in a homogeneously yellow coating having all of the azobenzene molecules back in the *trans* conformation. Next, a second pattern (tulip) was imprinted in the previously decorated/erased coating by a second mask illumination with UV light. In this way, different patterns can be reversibly imprinted in the coating. The imprinted patterns fade over time due to the limited half-life of the *cis* isomer upon storage at room temperature (∼20 °C). After 3 h, the optical pattern was still slightly visible, and after 18 h, the pattern almost fully disappeared as shown in Figure 8. Upon storage of the imprinted patterns at a lower temperature in a freezer (approximately −20 °C), the pattern remained visible for at least several weeks as a lower temperature increases the *cis* isomer lifetime enormously (Figure 9).^43^ This optical characteristic could be interesting as a time–temperature indicator, where the temperature history of frozen food or medicines could be monitored.

Interestingly, the incorporation of the azobenzene in the core or the shell did not have an effect on the water barrier performance of the coatings applied on paperboard (Figure S8). Cobb_10 min_ values of 1–3 g/m^2^ were found, indicating good water barrier performance and being similar to the Cobb values we previously reported for the coatings without azobenzene.^13^ For comparison, the uncoated paperboard had a Cobb_10 min_ value of 46 g/m^2^.^13^ Also, the isomerization state did not influence the barrier performance significantly.

## Conclusion

Two types of azobenzene-containing, photoresponsive, resin-stabilized waterborne dispersions were synthesized. The azobenzene moiety was incorporated into the polymer core by copolymerizing an azobenzene-functionalized methacrylate monomer (azo-in-core) or into the resin shell by esterifying the resin with an alcohol-functionalized azobenzene before emulsion polymerization (azo-in-shell). The dispersions were applied on glass and paperboard, to form photoresponsive coatings. The azobenzene moieties could be reversibly isomerized, in the polymer dispersions and coatings, using illumination with light of a specific wavelength. Interestingly, photoresponsive waterborne coatings show comparable photoresponsive properties when applied on either glass or paperboard. Also, the final properties of the coatings are independent of the synthesis route used during emulsion polymerization. Optical patterns can be imprinted in the coated paperboard by mask UV illumination and can be removed by exposing the patterns to blue light. The imprinted patterns fade slowly over hours at room temperature. Storing the patterned coatings in the freezer showed stable patterns for several weeks. The temperature-dependent stability of the imprinted patterns could be used to, e.g., monitor the temperature–time history of frozen goods. Furthermore, the good water barrier performance of the azobenzene-containing coatings on paperboard was unaffected, making them interesting for food packaging.

Our results reveal a class of photoresponsive azobenzene waterborne coatings that can be applied on different substrates and used for optical patterning, security, safety labeling, or sensing.

## Experimental Section

### Materials

Styrene (S, ≥99%), *n*-butyl acrylate (BA, ≥99%), 4-(dimethylamino)pyridine (DMAP), *N,N*′-dicyclohexylcarbodiimide (DCC), and *tert*-butyl hydroperoxide (70 wt % in water) were purchased from Sigma-Aldrich. Methacrylate- and alcohol-functionalized azobenzene (98.5% and 97.3%, respectively) were obtained from Synthon. The ASR solution, ammonium persulfate (APS), and sodium erythorbate were used as received from BASF. The alkali-soluble resin (ASR) was synthesized by copolymerization of acrylic acid with styrene, α-methylstyrene, and optionally a low percentage of an acrylic aliphatic ester. The weight-average molecular weight of the ASR is typically <10 kDa. A clear yellowish ASR solution with 30% solids and a pH of 7–8 was prepared by dissolving ASR (300 g) in water (620 g) via the addition of a 25% aqueous ammonium hydroxide solution (80 g). All chemicals were used as supplied. Deionized water was used throughout this research. The glass plates (3 cm × 3 cm) were cleaned with tissue paper with acetone and dried under an air flow. The paperboard substrate was kindly provided by Storaenso, type “Ensocard”. This is an uncoated bleached board having a thickness of 215 μm (170 g/m^2^).

### Synthesis of Azobenzene-Functionalized Resin

ASR (10.2 g) was ground in a mortar to a fine powder and dried in a vacuum line for 2 h. The powder was mixed with DMAP (0.5 g, 4.06 mmol) and alcohol-functionalized azobenzene (1.74 g, 4.88 mmol) in THF (40 mL), and this mixture was magnetically stirred until everything was dissolved and a clear yellow solution was obtained. Next, DCC was added (1.67 g, 8.36 mmol), and the reaction mixture was magnetically stirred for 72 h. The initial clear yellow solution turned cloudy after several hours as dicyclohexylurea formed. The suspension was filtered over a glass filter with a plug of Celite to remove the solids in the solution, followed by concentration of the filtrate by evaporation of the solvents in a rotary evaporator. A glassy yellow material was finally obtained after further drying in a vacuum line. The yellow crystals were dissolved in a 0.1 M NaOH aqueous solution (500 mL, pH 8–9), affording a slightly cloudy yellow solution. Filtration over a glass filter with Celite gave a clear yellow filtrate, which was further washed with chloroform (400 mL; three times, phase separation is slow) to remove any traces of solids. The product was finally precipitated upon acidification with concentrated HCl to a pH of 1–2, and the water layer was decanted. The solid azo-resin obtained was further washed with demi water (twice), dissolved in THF, and dried over solid Na_2_SO_4_. Filtration and concentration of the filtrate gave the azo-resin as a yellow-orange glassy product (8.9 g, 75%).

For gel permeation chromatography (GPC) characterization, free-standing films of the dispersions were dissolved in THF. The analysis was performed on a Shimadzu Prominence-I LC2030C 3D liquid chromatograph using unstabilized THF as the eluent (10 μL injection volume, 1 mg mL^–1^ sample in stabilized THF, filtered through a 0.2 μm filter). ^1^H NMR spectra were recorded on a Bruker Advance III HD 400 MHz instrument in either chloroform-*d* or DMSO-*d*_6_ (Sigma-Aldrich or TCI).

### Emulsion Polymerization (azo-in-core)

The dispersion with the azobenzene located in the polymer core (azo-in-core) was synthesized by semibatch emulsion polymerization. The polymerization was carried out in a 100 mL glass reactor equipped with a reflux condenser, an argon inlet, a feeding inlet, and a Teflon anchor type mechanical stirrer (200 rpm). The initial charge of water (6.00 g) and the ASR aqueous solution (19.50 g) was added to the reactor and heated to 84 °C under an argon atmosphere. The thermal initiator (APS, 0.15 g) was dissolved in water (0.60 g), and the mixture added to the preheated reactor. After being held for 3 min, the monomer blend of styrene (7.03 g), butyl acrylate (7.03 g), and methacrylate-functionalized azobenzene (0.44 g) was fed with a syringe pump and feed tube into the reaction mixture over 2 h. Subsequently, water (2.50 g) was flushed through the feed tube and the reaction mixture was kept at 84 °C for an additional 1 h. More water (3.75 g) was added, and the reaction mixture was cooled to room temperature under ambient conditions and finally filtered (50 μm mesh size). The dispersion was further used as obtained.

### Emulsion Polymerization (azo-in-shell)

The azobenzene-functionalized resin (6.51 g) was dissolved in water (13.43 g) with the addition of a 25% aqueous ammonium hydroxide solution (1.73 g) and stirred overnight to ensure complete dissolution. The dispersion with the azobenzene located in the resin shell (azo-in-shell) was also synthesized by semibatch emulsion polymerization using the same setup that was used for the azo-in-core dispersion. The initial charge of water (6.00 g) and the azobenzene-functionalized ASR aqueous solution (19.50 g) was added to the reactor and heated to 84 °C under an argon atmosphere. The thermal initiator (0.15 g) was dissolved in water (0.60 g) and added to the preheated reactor. After being held for 3 min, the monomer blend of styrene (7.25 g) and butyl acrylate (7.25 g) was fed with a syringe pump and feed tube into the reaction mixture over 2 h. Subsequently, water (2.50 g) was flushed through the feed tube and the reaction mixture was kept at 84 °C for an additional 1 h. Next, 70% aqueous *tert*-butyl hydroperoxide (0.13 g) in water (2.13 g) and sodium erythorbate (0.11 g) in water (2.12 g) were both slowly added over 3 h. More water (4.25 g) was added, and the reaction mixture was cooled to room temperature under ambient conditions and finally filtered (50 μm mesh size). The dispersion was further used as obtained.

### Dispersion Characterization

The dispersions were characterized by DLS (Zetasizer Nano Series, Malvern Instruments) to obtain the particle diameter. Dispersions were diluted with deionized water and filtered (GE Healthcare, glass microfiber filter with polypropylene housing, pore size of 2 μm) before measuring. The dry solid content (ASR included) was determined gravimetrically by the difference in the weight of the wet and dry dispersions. Approximately 1 g of the dispersion (wet) was applied on a filter paper, the dispersion dried in an oven at 140 °C for 20 min, and the solid residue weighed.

### Coating Preparation

Coatings on paperboard were prepared by applying ∼1.5 mL of the polymer dispersion on a paperboard substrate “Ensocard” (Storaenso, 18 cm × 30 cm) and making a “draw-down” using the bar coater (RK control coaster, speed level 8, 12 μm wet deposit wire bar). The coatings were dried at 60 °C for 1 h in a ventilated oven. Double-layered coatings were prepared to have better visual appearance. Hereto, a second layer was applied following the same procedure, after drying the first layer at 60 °C for 1 h. After being dried, the coatings were stored under ambient conditions for further characterization. The single layer had a thickness of ∼6 μm, and the thickness of the double-layer coating was ∼12 μm.

Coatings on glass were prepared in a manner similar to that used on paperboard using gap applicators of various thicknesses (10–90 μm). The coatings were dried on a hot plate at 60 °C for 1 h.

Free-standing films were prepared for GPC measurements by casting ∼0.50 g of the dispersion in a Teflon dish (diameter of 37 mm) and drying at room temperature (20 °C) overnight, followed by drying under vacuum for 15 h. The free-standing film was obtained by carefully removing the dried film from the Teflon dish.

### Coating Characterization

The coatings were characterized using ATR-FTIR (Varian 670 IR instrument equipped with a golden gate setup and Ge crystal). For easy comparison, the spectra were corrected with a simple baseline correction and normalized to the vibration peak at 2930 cm^–1^ using SpectraGryph version 1.2. UV–vis–NIR measurements of glass-coated samples were carried out on a PerkinElmer Lambda 750 UV–vis–NIR spectrophotometer equipped with a 150 mm integrating sphere containing a lead sulfide (PbS) and photomultiplier tube (PMT) detector. The background spectrum was recorded using a clean glass slide. The isomerization of azobenzene was triggered by shining for 1 min 365 nm (*I* = 45 mW/cm^2^) and 455 nm (*I* = 100 mW/cm^2^) light-emitting diode from Thorlabs. The half-life of *cis*-azobenzene was measured overnight, and every 15 min, a spectrum was recorded. The temperature was set to 25 °C to eliminate the influence of changes in environmental temperature. Topography images of the coating surface were made with atomic force microscopy (AFM) in ac mode (tapping mode). AFM imaging was performed with a Cypher ES Environmental atomic force microscope equipped with a closed cell and a heater-cool stage. The silicon probe (model AC160TSA) was manufactured by Olympus and purchased from Asylum Research. The probe has a 7 nm tip radius and a 14 μm tip height and operates with a spring constant *k* of 26 N/m and a frequency of 300 kHz. The methods for determining the water barrier performance were described previously.^12^

## Supporting Information Available

^1^H NMR spectra from the esterification reaction of the resin, calculations of the azobenzene concentration per polymer chain, influence of the isomerization state of azobenzene on particle size determined by DLS, absorption spectra of the azobenzene derivative dissolved in ethanol, fits used to determine the half-life of azobenzene in azo-in-core and azo-in-shell coatings, influence of UV light intensity of isomerization of the azo-in-core coatings, AFM and FTIR characterization of the coatings on paperboard, and water barrier performance of the azo-in-core coated paperboard (PDF)
