# Supplementary material for: Optical Patterning in Photoresponsive Azobenzene-Based Waterborne Coatings
Source: ACS Appl Opt Mater. 2022 Nov 17;1(1):403–11. doi: 10.1021/acsaom.2c00083 (PMC9903365; doi:10.1021/acsaom.2c00083)
Supplement: Supplementary file 1 — ot2c00083_si_001.pdf [file ot2c00083_si_001.pdf]

Supporting information

Optical patterning in photo-responsive azobenzene-based waterborne coatings

Sterre Bakker[a], Esmee de Korver[a], Michel Fransen[b], Esra Kamer[a], Gerald A. Metselaar[c], A. Catarina C. Esteves[d], Albert P.H.J. Schenning[a]**

[a] Laboratory of Stimuli-responsive Functional Materials and Devices, Department of Chemical Engineering and Chemistry, Eindhoven University of Technology, P.O. Box 513, 5600 MB Eindhoven, The Netherlands

[b] SyMO-Chem B.V., P.O. Box 513, 5600 MB Eindhoven, The Netherlands

[c] BASF Nederland B.V., Innovatielaan 1, 8447 SN Heerenveen, The Netherlands

[d] Laboratory of Physical Chemistry, Department of Chemical Engineering and Chemistry, Eindhoven University of Technology, P.O. Box 513, 5600 MB Eindhoven, The Netherlands

* Email: gerald.metselaar@basf.com / a.p.h.j.schenning@tue.nl

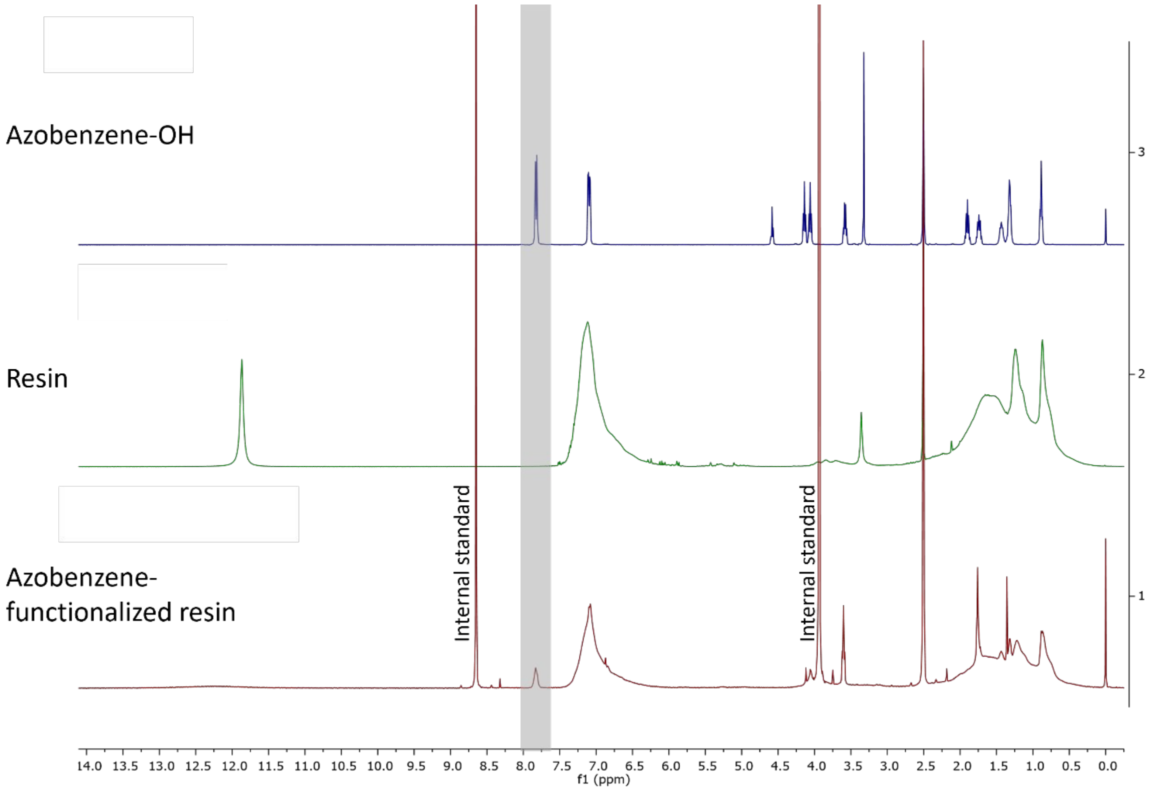

Figure S1. ^{1}H NMR of the unreacted resin and alcohol functionalized azobenzene and the esterificated product measured in DMSO.

Calculation of azobenzene concentration of polymer chain using ^{1}H NMR

The product polymer (**Figure S1**) shows signals at about 4.0 and 7.85 ppm that are not observed for the starting polymer resin and that are related to the presence of dye side chains. The product polymer ($8.828 \cdot 10^{-3}$ g; $2.207 \cdot 10^{-6}$ mol, assuming a molecular weight of 4 kg/mol) and internal standard (IS) trimethyl benzene-tricarboxylate ($4.745 \cdot 10^{-3}$ g; FW = 252.22 g/mol; $1.88 \cdot 10^{-5}$ mol) were mixed in DMSO-d6 and a ^{1}H-NMR spectrum was recorded. The three protons of the IS at about 8.7 ppm showed an integral of 3.00, while the four protons of the dye-moiety at

about 7.85 ppm showed an integral of 0.44. Thus, one proton corresponds to $1.88{\cdot}10^{-5}$ mol (eq. 1). In the product, the integral of 0.11 corresponds to one proton of the azobenzene incorporated in the product, which is a factor 9 of the IS. There is $2.069{\cdot}10^{-6}$ mol azobenzene incorporated in the product (eq. 2). Accordingly, every polymer chain on average has about 0.9 pendant dye moieties (eq. 3).

$$One\ proton = \frac{4.747 \cdot 10^{-3}}{252.22} = 1.88 \cdot 10^{-5}\ mol \qquad (1)$$

$$Azobenzene\ in\ product = \frac{1.88 \cdot 10^{-5}}{9} = 2.069 \cdot 10^{-6}\ mol \qquad (2)$$

$$Dye\ moieties\ per\ polymer\ chain = \frac{2.069 \cdot 10^{-6}}{2.207 \cdot 10^{-6}} = 0.9 \qquad (3)$$

Calculation of azobenzene concentration of polymer chain using UV-VIS

Figure S2 shows an overlay of UV spectra in THF of the starting resin (no absorbance at 360 nm), the azo-dye alcohol, the product material, and of a mixture of the starting resin and the azo-dye alcohol. For the azo-dye alcohol in THF we recorded a peak absorbance of 0.193 at 360 nm at a concentration of $3.63{\cdot}10^{-6}$ g (or $1.018{\cdot}10^{-8}$ mol) dye per gram of THF. For the product polymer, we found an absorbance of 0.163 at 360 nm at a concentration of $3.09{\cdot}10^{-5}$ g (or $7.725{\cdot}10^{-9}$ mol, assuming a molecular weight of product polymer of 4 kg/mol) polymer per gram of THF. Using the Lambert–Beer law (eq. 4) to calculate the extinction coefficient of the

pure azobenzene dissolved in THF (eq. 5). Assuming that the extinction coefficient does not change when the azobenzene is incorporated in the polymer, the azobenzene concentration in the polymer was calculated (eq. 6). Accordingly, every polymer chain has on average about 1.1 pendant dye groups (eq. 7).

$$A = \varepsilon \cdot l \cdot c \tag{4}$$

$$\varepsilon = \frac{A}{l \cdot c} = \frac{0.193}{1 * 1.018 \cdot 10^{-8}} = 1.895 \cdot 10^{7}\ mol\ cm^{-1}\ per\ gram\ THF \tag{5}$$

$$Azobenzene\ in\ product,\ c = \frac{A}{\varepsilon \cdot l} = \frac{0.163}{1.895 \cdot 10^{7} * 1} = 8.600 \cdot 10^{-9}\ mol\ per\ gram\ THF \tag{6}$$

$$Dye\ moieties\ per\ polymer\ chain = \frac{8.600 \cdot 10^{-9}}{7.725 \cdot 10^{-9}} = 1.1 \tag{7}$$

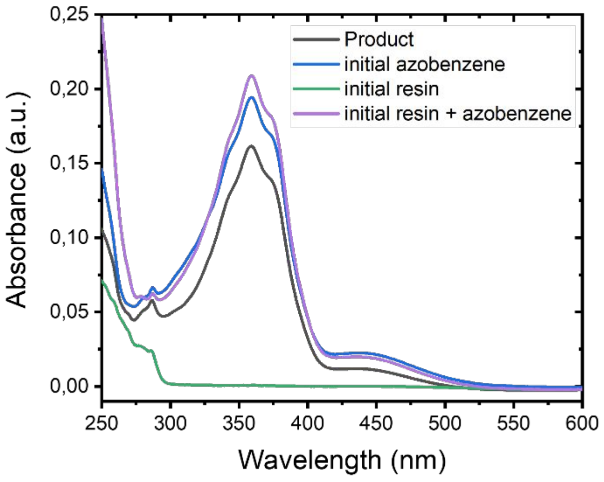

Figure S2. UV-visible measurements used for determining the azobenzene concentration in the resin.

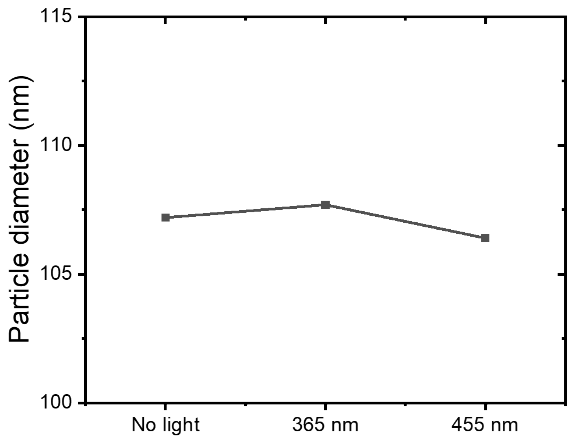

Figure S3. DLS measurement of the diluted azo-in-core dispersion before UV light, after UV light (365 nm) and after blue light (455 nm) illumination.

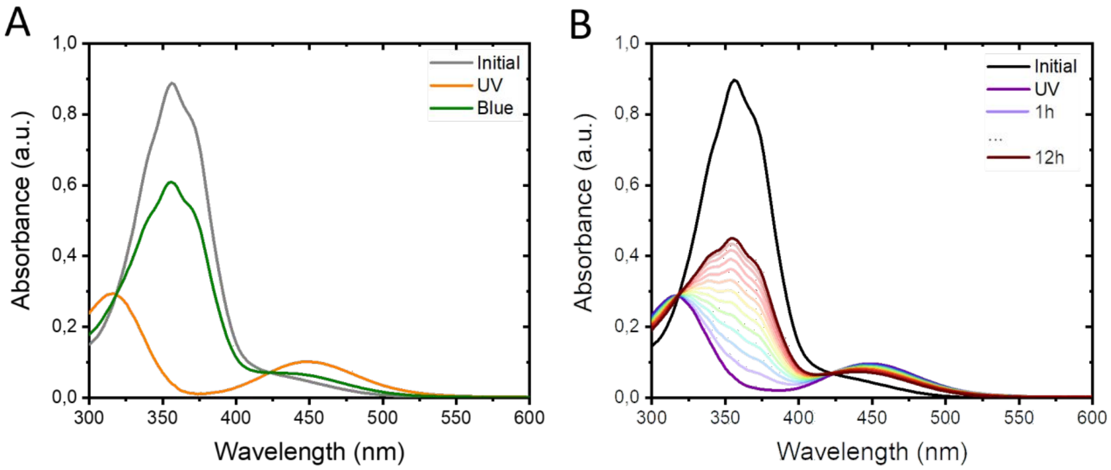

Figure S4. (A) UV-Visible spectra show the isomerization effect of the methacylate-azobenzene derivative dissolved in ethanol. (B) Half-life of the cis-azobenzene dissolved in ethanol was measured overnight.

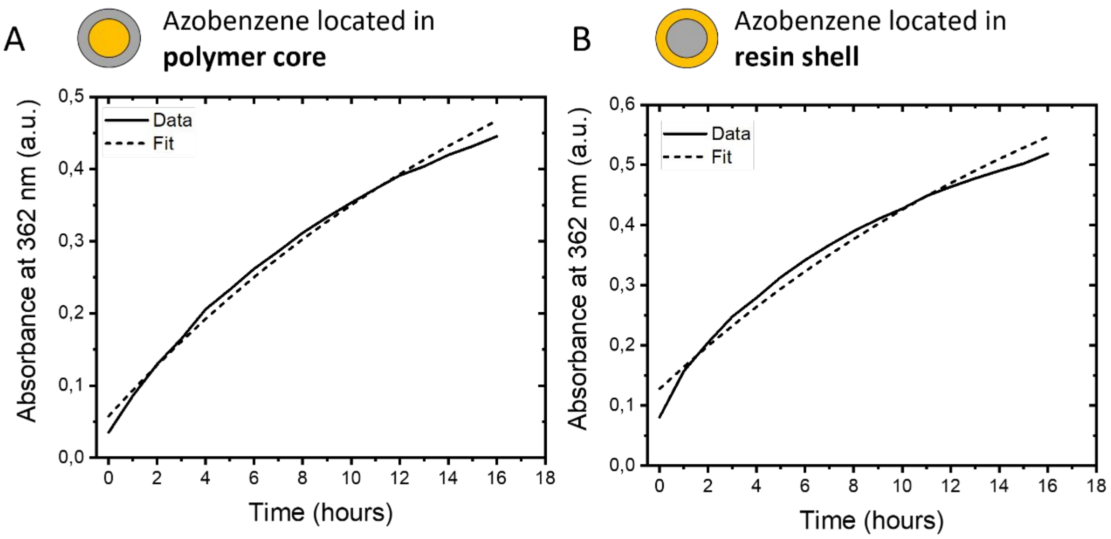

Figure S5. The absorbance at 362 nm versus time was plotted of the (A) azo-in-core and (B) azo-in-shell coatings on glass. Additionally, the half-life time was determined by the single exponential decay shown by the dashed lines. The standard deviation was calculated using equation 8 and have a value of 0.04 for the azo-in-polymer coating and 0.07 for azo-in-shell polymer coating.

$$\sigma = \sqrt{\sum |x_{fit} - x_{data}|} \qquad (8)$$

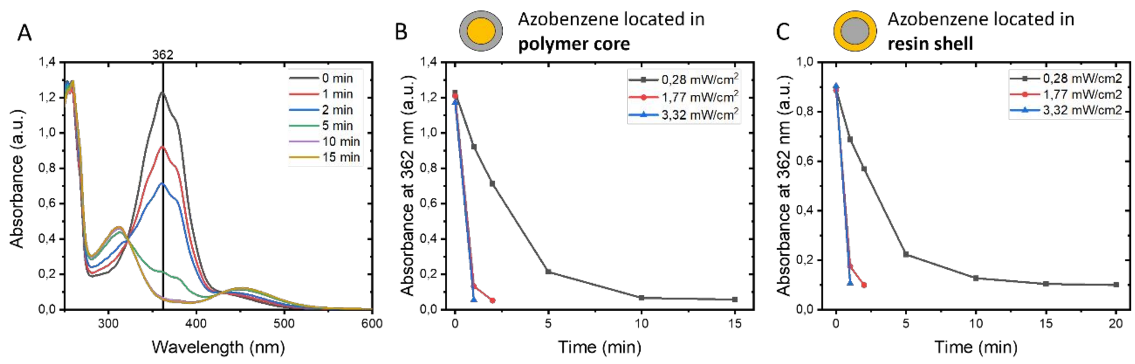

Figure S6. The effect of UV light intensity on the isomerization of the azobenzene in the coatings applied on glass. (A) The change in absorbance spectrum for the azo-in-core coating

on glass illuminated with 0.28 mW/cm^2 UV light over time. The decrease in absorbance at 362 nm was measured over time for several light intensities for the (B) azo-in-core and (C) azo-in-shell coatings applied on glass.

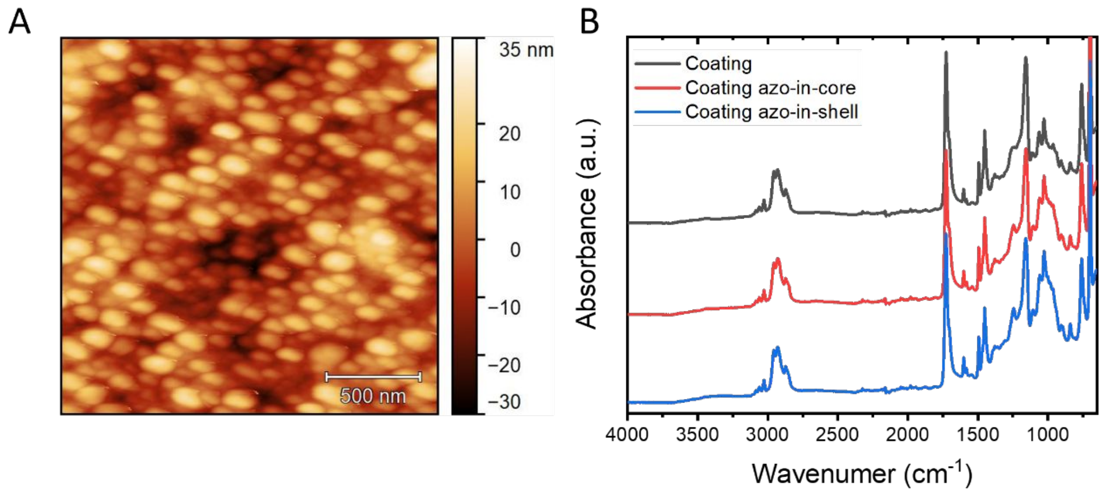

Figure S7. (A) AFM height image of the surface of the azo-in-core coating applied on paperboard. (B) ATR FTIR spectra of non-photo-responsive, azo-in core and azo-in-shell coatings on paperboard.

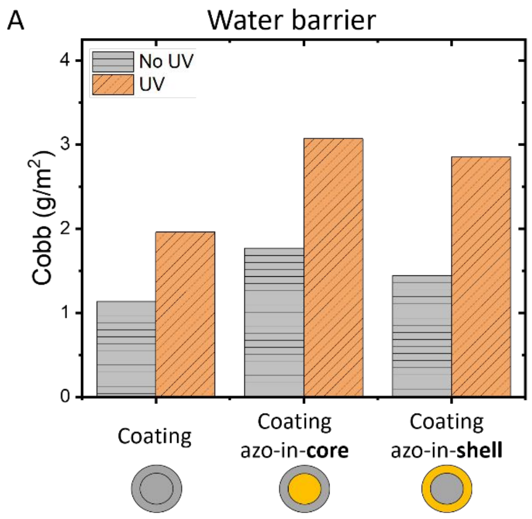

Figure S8. The water barrier performance in relation to the isomerization state of the azobenzene in the coating. Water barrier performance was determined by Cobb method after 10 minutes of water exposure for double-layer coatings on paperboard.
